# Analysis of the Structure-Function-Dynamics Relationships of GALT Enzyme and of Its Pathogenic Mutant p.Q188R: A Molecular Dynamics Simulation Study in Different Experimental Conditions

**DOI:** 10.3390/molecules26195941

**Published:** 2021-09-30

**Authors:** Anna Verdino, Gaetano D’Urso, Carmen Tammone, Bernardina Scafuri, Anna Marabotti

**Affiliations:** 1 Department of Chemistry and Biology “A. Zambelli”, University of Salerno, Via Giovanni Paolo II, 132, 84084 Fisciano (SA), Italy; averdino@unisa.it (A.V.); gaetano.durso@univ-rennes1.fr (G.D.); ctammone@unisa.it (C.T.); bscafuri@unisa.it (B.S.); 2 Interuniversity Center, ELFID—European Laboratory for Food Induced Diseases, University of Salerno, 84084 Fisciano (SA), Italy

**Keywords:** classic galactosemia, galactose-1-phosphate uridylyltransferase, rare diseases, galactose metabolism, molecular dynamics simulations

## Abstract

The third step of the catabolism of galactose in mammals is catalyzed by the enzyme galactose-1-phosphate uridylyltransferase (GALT), a homodimeric enzyme with two active sites located in the proximity of the intersubunit interface. Mutations of this enzyme are associated to the rare inborn error of metabolism known as classic galactosemia; in particular, the most common mutation, associated with the most severe phenotype, is the one that replaces Gln188 in the active site of the enzyme with Arg (p.Gln188Arg). In the past, and more recently, the structural effects of this mutation were deduced on the static structure of the wild-type human enzyme; however, we feel that a dynamic view of the proteins is necessary to deeply understand their behavior and obtain tips for possible therapeutic interventions. Thus, we performed molecular dynamics simulations of both wild-type and p.Gln188Arg GALT proteins in the absence or in the presence of the substrates in different conditions of temperature. Our results suggest the importance of the intersubunit interactions for a correct activity of this enzyme and can be used as a starting point for the search of drugs able to rescue the activity of this enzyme in galactosemic patients.

## 1. Introduction

**Scheme 1:**
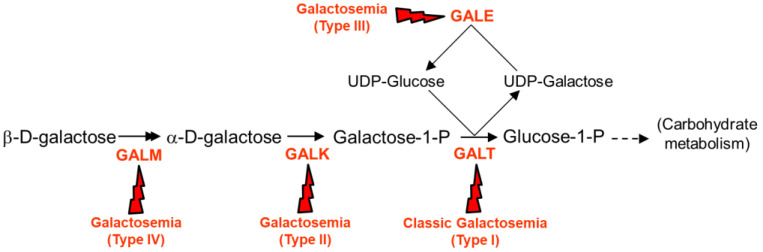
Leloir pathway of galactose metabolism. The enzymes catalyzing the different steps are galactose mutarotase (GALM), galactokinase 1 (GALK), galactose-1-phosphate uridylyltransferase (GALT), and UDP-galactose 4-epimerase (GALE). Different forms of galactosemia derive from the impairment of each enzyme, as represented in the scheme.

Galactose is an aldohexose sugar, C4-epimer of glucose. As suggested by its name (from the Greek term “galaktos”, which means “milk”), it is present mainly in milk and dairy products, bound to glucose to form the disaccharide sugar lactose, but it is also present in free or bound forms in many food plants [1]. Galactose metabolism has been named the “Leloir pathway” after the contribution of Luis Leloir to its elucidation [2], and it consists of four different steps in which galactose is converted into glucose-1-phosphate, which subsequently enters the glycolytic pathway [3] (Scheme 1).

Mutations in the gene coding for the third enzyme of this pathway, galactose-1-phosphate uridylyltransferase (GALT), which catalyzes the transfer of a uridine monophosphate (UMP) group from uridine diphosphate (UDP)-glucose to galactose 1-phosphate, thereby generating glucose 1-phosphate and UDP-galactose, cause the inborn error of metabolism known as “classic galactosemia” (OMIM: #230400). This disease manifests soon after birth if the newborn is exposed to galactose, with acute symptoms such as jaundice, feeding problems, failure to thrive, hepatocellular damage, bleeding, and possible death from *E. coli* sepsis. These neonatal signs are usually resolved with a (ga)lactose-restricted diet, but the dietary restriction does not avoid most galactosemic children to develop late complications, including developmental delays, problems in speech and in motor functions, and premature ovarian insufficiency in most affected females. To date, no treatments are available to avoid the insurgence of these complications [4].

**Figure 1:**
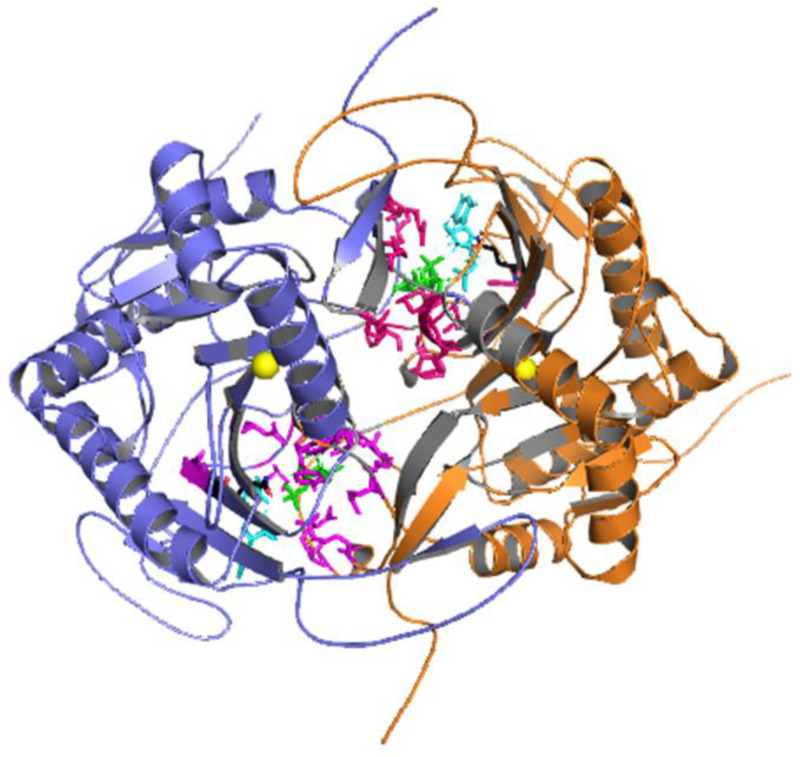
Structure of the model of the homodimeric GALT enzyme. Chain A is in blue, chain B in orange. The residues of the two active sites are represented in stick and colored in magenta, with the residue in position 188 in black. Galactose-1-phosphate (G1P) is in green and 5,6-dihydrouridine-5′-monophosphate (H2U) is in cyan. Zn ions are represented as yellow spheres.

Our research group is deeply involved in the characterization of the effects of GALT mutations at molecular level, by means of computational approaches. Most of these analyses were made in the past on the static structure of the models of the human enzyme obtained using as template the enzyme from *E. coli* [5,6,7,8], and more recently, on a new model of wild type enzyme [9] obtained using as template the crystallographic structure of the human enzyme carrying the polymorphism p.Asn314Asp, solved by McCorvie et al. [10]. GALT enzyme is a homodimeric protein, with two active sites located at the interface of the two subunits and formed by residues belonging to both subunits. In the structure solved by McCorvie et al., and in the model obtained from this structure, there are two Zn ions not involved in the catalysis, and two substrates bound in each active site: galactose-1-phosphate (G1P) and 5,6-dihydrouridine-5′-monophosphate (H2U) (Figure 1).

We used this last model as a starting point to model the structures of all the known galactosemia-associated mutants of the GALT enzyme and to perform a deep investigation of the effects of the mutations on the structure, function, and stability of the enzyme [9]. All results we collected in our past work were made freely available through the Web site http://www.protein-variants.eu/galactosemia/ (accessed on 29 September 2021), which also hosts similar analyses made on the other three enzymes of the Leloir pathway, associated with the other forms of galactosemia [9,11]. These analyses allowed us to identify the crucial role of several mutations in impairing the structural and functional features of the GALT enzyme and represent a wide effort to move from the molecular interpretation of the effect of mutations towards the link with phenotypic manifestations.

However, as the proteins are not static objects, we know that a considerable amount of information is lost if the effects of mutations cannot also be evaluated at a dynamic level. Molecular dynamics (MD) simulations allow the prediction of how every atom in a protein moves over time, and these simulations can capture important features of biomolecular processes. Then, they can be used both to interpret experimental results and to predict the impact of some perturbations (including addition or removal of a ligand and mutations) on biomolecules, thus guiding future experimental work [12]. In particular, computational methods to elucidate intra- or intersubunit interactions or communication patterns essential for functional activities were used to characterize those molecular features, which can be targeted for the development of therapeutic approaches (see, for example, [13,14]). In the past, we performed MD for a limited simulation time on selected mutations of GALT (some of which were novel at that time) [15]. We present here MD simulation studies on the structure and dynamics of the human wild-type GALT enzyme (wtGALT) and of the mutant p.Gln188Arg (the most frequent mutation and the one associated to the most severe galactosemic phenotype) [4] in different conditions. We dissect more deeply the perturbations that this mutation, associated to classic galactosemia, can induce on protein structure and dynamics. In particular, MD simulations have been performed both in the presence and in the absence of the two ligands in the active site of the enzyme at two different temperatures: 310 K, corresponding to the normal body temperature, and 334 K. In this last case, we would like to simulate an environment that favors the destabilization of the enzyme, to predict the molecular effects (if any) of this destabilization, particularly at the level of intersubunit interactions. 

This study is a part of an effort to understand if the use of pharmacological chaperones (small molecules that, by binding to specific target proteins, can stabilize their native conformation or correct their misfolding [16]) may be practicable also in the case of classic galactosemia, as suggested in the past [17]. In our associate publication [18] we focus on the computational studies of the predicted molecular interactions between arginine, a putative pharmacochaperone that was found able to improve the activity of p.Gln188Arg and other GALT mutants in a prokaryotic model of classic galactosemia [19], and both the wild-type and mutant p.Gln188Arg enzymes.

## 2. Results and Discussion

### 2.1. Analysis of MD Simulations at 310 K

The simulations performed on both wtGALT and p.Gln188Arg at 310 K (the normal human body temperature), in the presence and in the absence of the substrates, were analysed to check that no major perturbations affected them. The analysis of the energetic components (including total energy, kinetic energy component, potential energy component, pressure, temperature, volume, and density), of the minimum distance of periodic images, and of the root mean square deviation (RMSD) of atom distances, showed that both systems quickly reached the stabilization (see Supplementary Files S1–S4).

**Figure 2:**
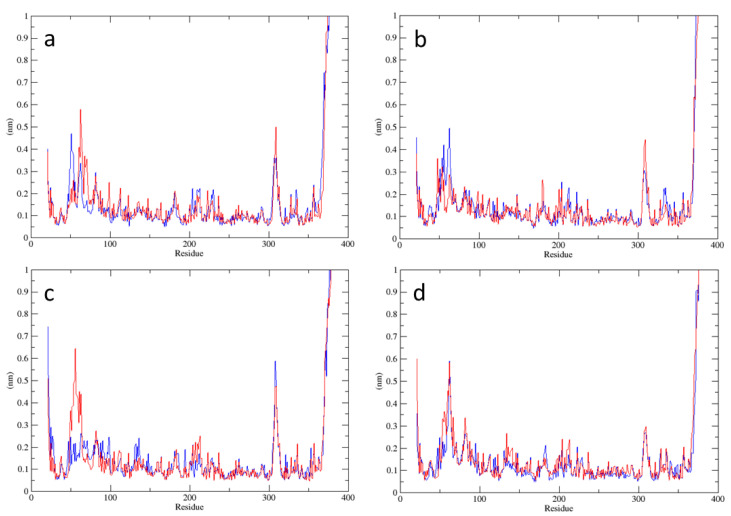
RMSF analysis for simulations at 310 K of: (**a**): wtGALT; (**b**): p.Gln188Arg; (**c**) wtGALT + G1P + H2U; (**d**) p.Gln188Arg + G1P + H2U. Blue lines represent RMSF fluctuation of chain A, red lines of chain B.

A slight difference in the global structural features of these two systems, in the absence of substrates, can be detected with the analysis of the root mean square fluctuation (RMSF), which shows a slightly enhanced flexibility of the mutant in the zone of residues ~50–70 (including segment 50–60 formed by very conserved residues at the intersubunit interface, some of which are also involved in substrate interactions), ~300–320 (a long loop including three conserved His residues (His301, His319, His321) forming the Zn binding site), and marginally in the zone of mutation. The fluctuation, especially for segment 300–320, is not identical in the two chains (Figure 2a,b). In the presence of the substrates, the RMSF graph shows for both systems an enhancement of the flexibility around residues 50–70 and, for the mutant, a decrease in the flexibility of the segment between residues 300–320 with respect to the systems in the absence of the ligands (Figure 2c,d), in particular for one of the two chains. Thus, it appears that the presence of the substrates has a different effect on the local flexibility of wtGALT with respect to p.Gln188Arg.

The analysis of the content of secondary structures (Figure S1a,b) did not highlight the main differences between the two systems in the absence of the ligands. In the presence of the substrates, instead, the mutant enzyme has a slight increased content in less regular structures, such as the π-helix (indicated as 5-helix in the graph) (Figure S1c,d). Once again, in the presence of the substrates, the mutant enzyme shows a perturbation that could be diagnostic of the alteration of its structural features due to the mutation.

During the simulations, the radius of gyration remained stable and practically identical for all systems (Figure S2, panels a–d). The predicted solvent-accessible surface area (SASA) was similar for systems in the absence of substrates, with a small decrease (from 310 to 290 nm^2^) in both cases and a slightly more accentuated decreasing trend for the mutant enzyme (Figure S3, panels a,b). In the simulation in the presence of the substrates, the SASA of wtGALT is fluctuating around an average value of 305 nm^2^, whereas in p.Gln188Arg it shows a marked decreasing tendency along the trajectory from about 320 nm^2^ to 300 nm^2^ (Figure S3, panels c,d).

**Figure 3:**
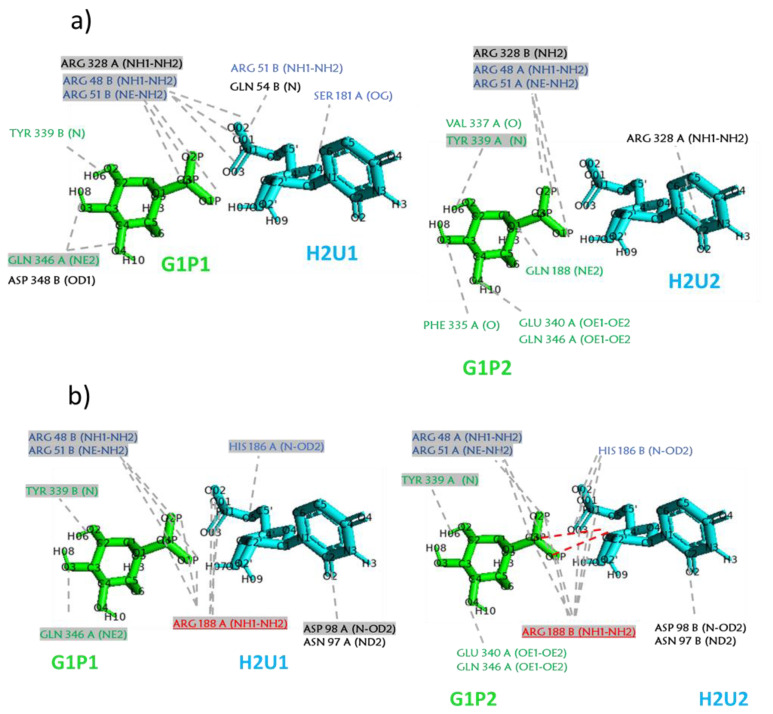
Interactions between enzyme and ligands in simulations at 310 K. (**a**) wtGALT; (**b**) p.Gln188Arg. Panels on the left indicate the ligands of the active site 1 (formally belonging to subunit A), panels on the right indicate the ligands of the active site 2 (formally belonging to subunit B). Grey background indicates interactions that persist for more than 50% of the simulation time. Grey dashed lines indicate H-bonds; residues underlined are also able to form salt bridges. Red dashed lines indicate interactions between the ligands.

The analysis of the interactions between the enzymes and the ligands present in both active sites show that both ligands are stably bound to the proteins during the simulations (Figure S4a–d). Both in wtGALT and in p.Gln188Arg, G1P interacts with residues belonging to both chains, mainly with residues Arg48 and Arg51 and less stably with residues belonging to the segment 330–340 of the protein. In wtGALT, H2U interacts with several residues, but does not show persistent interactions. On the contrary, in p.Gln188Arg, H2U shows persistent interactions with His 186 and Arg188. In particular, with this last residue, both hydrogen bonds and salt bridges are predicted to occur. The interactions of the substrates are not exactly symmetrical in both active sites (Figure 3).

To verify if the introduction of the mutation p.Gln188Arg in the protein could destabilize its quaternary assembly, we analysed the H-bonds present at the intersubunit interface. The results are shown in Table 1. Many stable interactions listed in Table 1 were not detected in the static models of the enzymes [9,10], but they are preserved during the simulations and are present in both systems. The average number of intersubunit H-bonds calculated per each timeframe is slightly higher in p.Gln188Arg than in wtGALT, but, interestingly, when the ligands are bound to the enzymes, the number of stable H-bonds is consistently higher in wtGALT than in p.Gln188Arg (Table 1). This suggests that the ligands exert a stabilizing effect on the quaternary assembly of the wild-type enzyme and that this effect is lost in the mutant.

We also calculated the intersubunit salt bridges present in the different simulations and found that their number is slightly higher in the mutant enzyme with respect to wtGALT in the absence of ligands, whereas the opposite is true in the presence of the ligands (Table 2), as it happens for H-bonds (Table 1). However, considering the low number of stable salt bridges detected during the simulations, it is not possible to deduce if this difference is significant. A salt bridge between Asp113 of chain A and Arg228 of chain B visible in the static model, is conserved in the simulations. Instead, another intersubunit salt bridge that is visible in the starting structure (His114B-Glu220A) [9,10] is not preserved during all the simulations. Additional salt bridges between the Glu58 of chain A and the Arg333 of chain B and between the Asp98 of chain A and the Arg51 of chain B are stably present in p.Gln188Arg only in the absence of substrates.

From these analyses it is possible to infer that the presence of the mutation is able to perturb the correct intersubunit interactions present in wtGALT, but this perturbation is more evident when the ligands are bound in the active site. Although the length of the simulation cannot allow for the detection of a complete destabilization of the enzyme, these effects appear to confirm what it was deduced from the analysis of the static structure, i.e., the replacement of Gln188 by Arg not only impairs the enzymatic activity, but also the stability of the quaternary assembly, and probably, the two effects are correlated.

### 2.2. Analysis of MD Simulations at 334 K

We simulated the two systems at higher temperature, near their T_m_ [10,20] in order to induce a perturbation of the structural features of these two systems and see if there are different behaviors at molecular level. The quality checks of the trajectories obtained at higher temperature confirmed the good stabilization and the correct behavior of the systems during the simulations (Supplementary Files S5–S8). 

**Figure 4:**
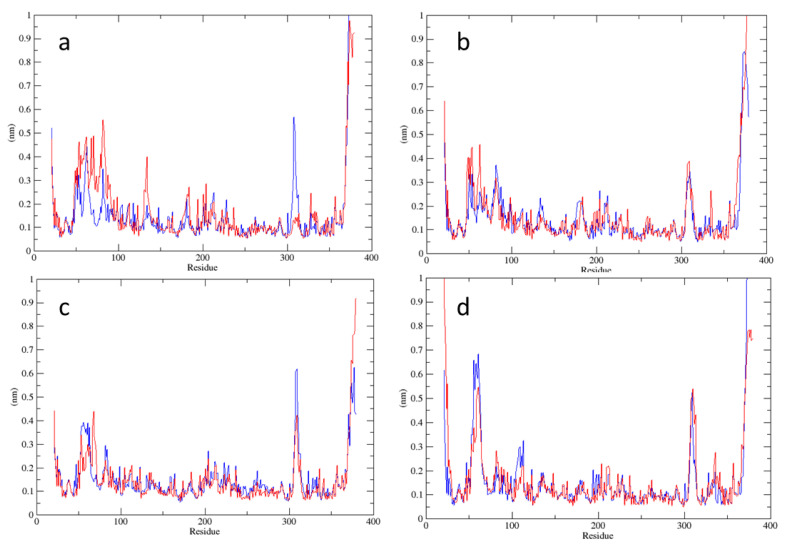
RMSF analysis for simulations at 334 K of: (**a**): wtGALT; (**b**): p.Gln188Arg; (**c**) wtGALT + G1P + H2U; (**d**) p.Gln188Arg + G1P + H2U. Blue lines represent RMSF fluctuation of chain A, red lines of chain B.

The RMSF graphs of the simulations in the absence of the substrates (Figure 4a,b) do not reveal a great enhancement in the flexibility of the protein structure with respect to the equivalent systems at 310 K. Again, the most fluctuating parts of the enzymes are the segments including residues ~50–70 and ~300–320, and again, the fluctuation is not identical in the two subunits. Differently from the simulations at 310 K, in the mutant enzyme, the presence of the ligands enhances the fluctuation in these segments, with respect to the simulation at 334 K in the absence of ligands (Figure 4c,d). 

The analysis of secondary structures (Figure S1, panels e–h) reveals the formation of more irregular structures such as the π-helix (indicated as 5-helix in the graph produced by DSSP algorithm) in wtGALT, and a small increase in the number of disordered structures such as coils in p.Gln188Arg. The presence of the ligands seems to stabilize the secondary structures, since in wtGALT the irregular structures such as π−←←helices are no longer detected, and the number of residues in the coils is reduced in p.Gln188Arg; however, this last system is still more perturbed given that the presence of the π-helices is still detectable. Thus, as in the case of the simulations at lower temperature, in the mutant enzyme, the presence of the substrates is not able to completely rescue the destabilization. 

**Figure 5:**
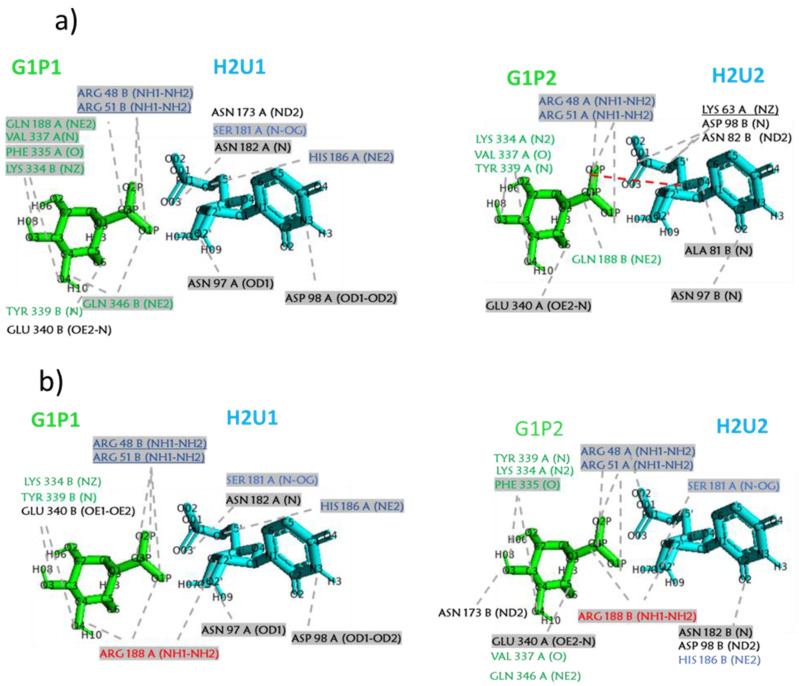
Interactions between enzyme and ligands in simulations at 334 K. (**a**) wtGALT; (**b**) p.Gln188Arg. Panels on the left indicate the ligands of the active site 1 (formally belonging to subunit A), panels on the right indicate the ligands of the active site 2 (formally belonging to subunit B). Grey background indicates interactions that persist for more than 50% of the simulation time. Grey dashed lines indicate H-bonds; residues underlined are also able to form salt bridges. Red dashed lines indicate interactions between the ligands.

Even at higher temperature, the ligands in the active site remain stably associated with the enzymes (Figure S4e–h). The detailed interactions between the ligands and the residues of the active site are reported in Figure 5. The persistent interactions between G1P and Arg48 and Arg51 predicted at 310 K, both in wtGALT and p.Gln188Arg are conserved even at this higher temperature, as well as the persistent interactions between H2U and Arg188, via hydrogen bonds and salt bridges. Therefore, also at high temperature it is possible to suppose that the mutant residue Arg188 is able to interfere with the correct enzymatic activity of GALT. Again, the interactions found in the two active sites are not identical. 

The analysis of the radius of gyration (Figure S2, panels e–h) does not highlight the main differences between the two proteins, whereas the analysis of the SASA (Figure S3, panels e–f) shows that p.Gln188Arg has a higher value of this parameter (average value between 330 and 340 nm^2^ compared to the average value between 290 and 300 nm^2^ in wtGALT), indicating that the mutant protein, at higher temperature, tends to expose a higher area to the solvent, and this effect is clearly more visible at high temperature. In the presence of the ligands, the SASA is slightly lower in wtGALT than in p.Gln188Arg and, for the mutant, is lower than the value predicted in the absence of the ligands (Figure S3, panels g–h), reaching the same value of the simulations at 310 K.

The analysis of the interface interactions shows that, in the simulations in the absence of the ligands at higher temperatures, the average number of H-bonds per timeframe are practically unchanged in both systems with respect to the simulations at 310 K (Table 3). Contrarily to what happens in the systems at 310 K, the presence of the ligands in wtGALT does not increase this parameter, suggesting that, at a higher temperature, they are no longer able to promote a further stabilization of the intersubunit interface. Instead, in p.Gln188Arg, this parameter is decreased, as it happens at body temperature, showing that the mutation still destabilizes this interface. In addition, at high temperature, many stable H-bond interactions, including some that are not detectable in the static structures, are formed during the simulations, and their number is higher in the absence than in the presence of the ligands, in both systems. The residues involved in these interactions are essentially the same predicted to interact at body temperature. 

The number of salt bridges is identical both between the two systems in the absence of ligands and between the two systems in the presence of ligands and is lower in the latter case (Table 4). Again, the number of these interactions is so low that it is not possible to state if this difference is significant or not. In all systems, the salt bridge between Asp 113 and Arg 228 is conserved, whereas the interaction between the Glu58 of chain A and the Arg333 of chain B is lost in the presence of the substrates, as it was the case for simulations at 310 K (Table 2).

From these results it appears that at 334 K both systems are not dramatically perturbed, although some differences are present especially in the flexibility of the most mobile segments and in the secondary structures. The temperature seems not to perturb drastically the quaternary assembly of the enzymes, but the ability of the substrates to stabilize especially the intersubunit H-bonds seems to be lost in wtGALT at higher temperatures, and in p.Gln188Arg in all conditions.

## 3. Materials and Methods

### 3.1. Starting Structures

We have used as a starting point the models of wtGALT and of the mutant p.Gln188Arg obtained as described previously [9], derived from the crystallographic structure of human GALT enzyme (PDB code: 5IN3) [10]. Both models contain the ligands galactose-1-phosphate (G1P) and 5,6-dihydrouridine-5′-monophosphate (H2U) that were visible in the crystallographic structure, as well as the Zn ions. However, during the modelling process, the covalent bond between H2U and the residue His186 has not been modelled, and the structure of this ligand in both cases has been corrected in order to restore the normal phosphate group by using Chimera [21]. This has also avoided problems in the parameterization of this anomalous bond in the following steps. The phosphate groups of the ligands were considered in their charged (deprotonated) form throughout the simulations.

### 3.2. MD Simulations and Analysis

MD simulations were performed on two supercomputers available at CINECA Consortium, the largest Italian computing centre (Casalecchio di Reno, Italy): GALILEO, an IBM NeXtScale cluster, with 1022 36-core compute nodes, each containing 2 × 18-cores Intel Xeon E5-2697 v4 (Broadwell) at 2.30 GHz, with Linux Infiniband architecture; MARCONI, a Lenovo NeXtScale Cluster, with 3188 48-core computing nodes, each containing 2 × 24-cores Intel Xeon 8160 (SkyLake) at 2.10 GHz, with Intel OmniPath architecture. The access to these computational resources was made available thanks to ELIXIR-IT [22]. The package used for the MD simulations was GROMACS 2018.3 [23]. The force field used throughout the simulation was AMBER99SBildn [24,25], and the packages ANTECHAMBER and ACPYPE [26,27] were used according to their instructions to calculate the correct topology for the ligands. Each of the four starting systems (wtGALT; wtGALT + ligands; p.Gln188Arg; p.Gln188Arg + ligands) was included in a cubic box centered on the protein, with a distance of 1.2 nm from it, filled with water (using the TIP4P model [28]), and neutralized with chloride/sodium ions. The four systems were first minimized by applying steepest descent minimization, setting the cut-off for short-range electrostatic and van der Waals interactions to 1.2 nm, and using the grid method to determine the neighbor list. Minimization stopped when the maximum force reached a value lower than 10.0 kJ/mol/nm. Equilibration steps with position-restrained MD simulations (by applying position restraints to all the heavy atoms of the protein to equilibrate the solvent) were run first in NVT (constant number of particles, volume, and temperature) conditions for 100 ps and subsequently in NPT (constant number of particles, pressure, and temperature) conditions for 1000 ps. For the NVT equilibration, the V-rescale thermostat [29] was applied; for NPT equilibration, the Berendsen barostat [30] was added to keep the pressure constant at 1.0 bar. At the end of the equilibration, for each system, we performed 200-ns-long MD simulations in NPT conditions, at a temperature of 310 K or 334 K. For the production runs, the Berendsen barostat was replaced with the Parrinello–Rahman barostat [31]. The other parameters selected for the production simulations were the leap-frog algorithm [32] for integrating Newton equations of motion; the LINear Constraint Solver (LINCS) algorithm [33] to constrain bonds; Verlet [34] as cutoff scheme in the neighbor searching section; Particle Mesh Ewald (PME) method [35] to handle long-range electrostatic interactions.

At the end of the simulations, the trajectories were analyzed using GROMACS analysis tools. The obtained results were plotted by using XMGrace software (https://plasma-gate.weizmann.ac.il/Grace/; last accessed 24 September 2021). The analyses of the content in the secondary structures were performed by using DSSP, the de facto standard algorithm for secondary structure assignment [36]. The percentage of the existence of intersubunit hydrogen bonds was calculated with the Perl script plot_hbmap.pl provided by Prof. Justin Lemkul (https://www.thelemkullab.com/; last accessed 24 September 2021), whereas the percentage of existence of salt bridges was performed by using an in house developed Perl script on 4000 PDB files extracted from the trajectory using GROMACS commands. We summed up the percentages of the existence of hydrogen bonds or salt bridges involving two residues, and only when this sum was equal or higher than 50, did we consider these two residues as stably interacting during the simulation.

## 4. Conclusions

p.Gln188Arg is the most common pathogenic mutation of the GALT enzyme. This mutation is associated with the most severe phenotype and to a poor outcome of the classic galactosemia disease. This mutant enzyme has no or barely any detectable enzymatic activity in the erythrocytes and liver of homozygous patients, and less than 50% activity in heterozygous individuals [4]. It has been proposed that this partial dominant effect could be related to the perturbation of the molecular interface between the two subunits forming the quaternary structure of the enzyme, considering that Gln188 is not only a residue of the active site but also a residue located at the interface between the two subunits [5,9,10].

In this work, we have performed a thorough analysis of the structural effects that mutation p.Gln188Arg can induce on GALT enzyme, particularly from a dynamics point of view. From our results at body temperature (310 K), it appears that the negative effects of the mutation on the intersubunit interactions are more evident in the presence of the ligands. Indeed, the wild-type enzyme bound to the substrates shows an increased number of intersubunit H-bonds, most of which are not predicted in the static structure but are formed and persist during the MD simulations. On the contrary, the mutant p.Gln188Arg shows a marked decrease of the number of intersubunit H-bonds in the presence of the substrates. The number of intersubunit salt bridges is very small and it is not possible to infer if the variations detected are significant or not, but their trend in both wtGALT and p-Gln188Arg is analogous to that of H-bonds. It is also interesting to see that, despite this being an homodimeric enzyme, the flexibility of the same segments in the two subunits is not of the same extent. This is intriguing, considering that the zones characterized by higher flexibility are either at the subunit interface or are involved in the stabilization of Zn, which is considered to have an important structural role for this enzyme [9,10].

The higher temperature used to perturb our systems (334 K) seems to have few effects on the overall structure of the enzyme, but in these simulations, it is also possible to see that the mutation perturbs the quaternary assembly of the enzyme. 

Overall, our simulations confirm the importance of the intersubunit interactions of GALT for its correct functioning and suggest that their preservation in the mutant could improve the functioning of the enzyme, thereby rescuing, at least partially, its activity. 

## Figures and Tables

**Table 1:** Couples of residues involved in stable intersubunit H-bonds in simulations at 310 K. Dark gray background: intersubunit interactions identified in the initial models and conserved throughout the simulations; bold: stable interactions present in all systems. Couples of residues are considered to have a stable H-bond interaction when the sum of % of existence of the H-bonds between the two residues is ≥50.

wtGALT	p.Gln188Arg	wtGALT + Ligands	p.Gln188Arg + Ligands
Average Number of H-Bonds per Timeframe: 27	Average Number of H-Bonds per Timeframe: 29	Average Number of H-Bonds per Timeframe: 30	Average Number of H-Bonds per Timeframe: 25
**ILE32A-LYS120B**	**ILE32A-LYS120B**	**ILE32A-LYS120B**	**ILE32A-LYS120B**
**ILE32B-LYS120A**	**ILE32B-LYS120A**	**ILE32B-LYS120A**	**ILE32B-LYS120A**
**TYR34A-GLN118B**	**TYR34A-GLN118B**	**TYR34A-GLN118B**	**TYR34A-GLN118B**
**TYR34B-GLN118A**	**TYR34B-GLN118A**	**TYR34B-GLN118A**	**TYR34B-GLN118A**
**ILE198A-ALA343B**	**ILE198A-ALA343B**	ARG48A-PHE99B	ARG48A-PHE99B
ILE198B-ALA343A	ARG48A-PRO100	**ILE198A-ALA343B**	ASP197A-GLN344B
HIS301A-LEU342B	HIS301A-LEU342B	ILE198B-ALA343A	SER45A-ALA101B
ASP197A-GLN334B	ASP197A-GLN334B	HIS47B-PRO100A	**ILE198A-ALA343B**
ARG48A-PHE99B	GLN30A-GLN103B	ASP197B-GLN344A	HIS47B-PRO100A
SER45A-ALA101B	GLN30A-ALA122B	**TRP41A-ASP197B**	**TRP41A-ASP197B**
GLN30A-GLN103B	**TRP41A-ASP197B**	TRP41B-ASP197A	TRP41B-ASP197A
GLN30A-ALA122B	HIS47A-PRO100B	GLN30B-GLN103A	GLN30B-GLN103A
**TRP41A-ASP197B**	**ARG228B-ASP113A**	GLN30B-ALA122A	GLN30B-ALA122A
HIS47A-PRO100B	ARG333B-GLU58A	ARG228A-ASP113B	ARG228A-ASP113B
**ARG228B-ASP113A**	ARG228A-ASP113B	**ARG228B-ASP113A**	**ARG228B-ASP113A**
ARG333B-GLU58A	ARG201A-ASP39B	GLY338A-SER297B	GLY338A-SER297B
SER45B-ALA101A	GLN103A-GLN30B	SER45B-ALA101A	SER45B-ALA101A
ARG51B-ASP98A	GLY338B-SER297A	GLY338B-SER297A	GLN224A-HIS114B
		ARG51B-ASP98A	ARG48B-PHE99A
		GLN30A-GLN103B	
		GLN30A-ALA122B	
		ARG48B-PHE99A	

**Table 2:** Couples of residues involved in stable intersubunit salt bridges in simulations at 310 K. Couples of residues are considered to have a stable salt bridge interaction when the sum of % of existence of the salt bridges between the two residues is ≥50.

wtGALT	p.Gln188Arg	wtGALT + Ligands	p.Gln188Arg + Ligands
GLU58A-ARG333B	GLU58A-ARG333B		
	ASP113B-ARG228A	ASP113B-ARG228A	ASP113B-ARG228A
ASP113A-ARG228B	ASP113A-ARG228B	ASP113A-ARG228B	ASP113A-ARG228B
ASP98A-ARG51B	ASP98A-ARG51B	ASP98A-ARG51B	

**Table 3:** Couples of residues involved in stable intersubunit H-bonds in simulations at 334 K. Dark gray background: intersubunit interactions identified in the initial models and conserved throughout the simulations; bold: stable interactions present in all systems. Couples of residues are considered to have a stable H-bond interaction when the sum of % of existence of the H-bonds between the two residues is ≥50.

wtGALT	p.Gln188Arg	wtGALT + Ligands	p.Gln188Arg + Ligands
Average Number of H-Bonds per Timeframe: 27	Average Number of H-Bonds per Timeframe: 28	Average Number of H-Bonds per Timeframe: 27	Average Number of H-Bonds per Timeframe: 26
**ILE32A-LYS120B**	**ILE32A-LYS120B**	**ILE32A-LYS120B**	**ILE32A-LYS120B**
**ILE32B-LYS120A**	**ILE32B-LYS120A**	**ILE32B-LYS120A**	**ILE32B-LYS120A**
**TYR34A-GLN118B**	**TYR34A-GLN118B**	**TYR34A-GLN118B**	**TYR34A-GLN118B**
ASP197A-GLN344B	TYR34B-GLN118A	TYR34B-GLN118A	TYR34B-GLN118A
ILE198A-ALA343B	ASP197A-GLN344B	ARG48A-PHE99B	ARG48A-PHE99B
ARG48A-PHE99B	ILE198A-ALA343B	SER45B-ALA101A	SER45A-ALA101B
SER45A-ALA101B	GLN30A-GLN103B	ASP197A-GLN344B	ASP197B-GLN344A
HIS47B-PRO100A	GLN30A-ALA122B	ILE198A-ALA343B	ILE198B-ALA343A
HIS301B-LEU342A	**ARG228A-ASP113B**	HIS301A-LEU342B	HIS301B-LEU342A
**TRP41B-ASP197A**	**ARG228B-ASP113A**	GLY338A-SER297B	GLY338A-SER297B
**GLY338B-SER297A**	**TRP41B-ASP197A**	**GLY338B-SER297A**	**GLY338B-SER297A**
ARG333B-GLU58A	**GLY338B-SER297A**	ARG48B-PHE99A	**ARG228A-ASP113B**
GLN103B-GLN30A	ARG333A-GLU58B	GLN30B-GLN103A	**ARG228B-ASP113A**
GLN30A-ALA122B	SER297B-VAL337A	GLN30B-ALA122B	ARG48B-PHE99A
**ARG228B-ASP113A**	TRP41A-ASP197B	TRP41A-ASP197B	TRP41A-ASP197B
**ARG228A-ASP113B**	GLN169B-LEU342A	**TRP41B-ASP197A**	**TRP41B-ASP197A**
GLN30B-GLN103A	SER45B-ALA101A	**ARG228A-ASP113B**	ALA122A-GLN30B
GLN30B-ALA122A	GLN56A-PRO59B	**ARG228B-ASP113A**	
ARG201B-ASP39A	ARG48B-GLN103A		
HIS47A-PRO100B			
GLY338A-SER297B			

**Table 4:** Couples of residues involved in stable intersubunit salt bridges in simulations at 334 K. Couples of residues are considered to have a stable salt bridge interaction when the sum of % of existence of the salt bridges between the two residues is >50.

wtGALT	p.Gln188Arg	wtGALT + Ligands	p.Gln188Arg + Ligands
GLU58A-ARG333B	GLU58A-ARG333B		
ASP113B-ARG228A	ASP113B-ARG228A	ASP113B-ARG228A	ASP113B-ARG228A
ASP113A-ARG228B	ASP113A-ARG228B	ASP113A-ARG228B	ASP113A-ARG228B

## Data Availability

Data is contained within the article or supplementary material.

## Supplementary Materials

Figure S1: DSSP analysis, Figure S2: Radius of gyration, Figure S3: SASA, Figure S4: Pair distance between enzyme and substrates, File S1: Quality check for wtGALT at 310 K, File S2: Quality check for p.Gln188Arg at 310 K, File S3: Quality check for wtGALT + ligands at 310 K, File S4: Quality check for p.Gln188Arg + ligands at 310 K, File S5: Quality check for wtGALT at 334 K, File S6: Quality check for p.Gln188Arg at 334 K, File S7: Quality check for wtGALT + ligands at 334 K, File S8: Quality check for p.Gln188Arg + ligands at 334 K.

## Sample Availability

Not available.
