# Supplementary material for: Analysis of the Structure-Function-Dynamics Relationships of GALT Enzyme and of Its Pathogenic Mutant p.Q188R: A Molecular Dynamics Simulation Study in Different Experimental Conditions
Source: Molecules. 2021 Sep 30;26(19):5941. doi: 10.3390/molecules26195941 (PMC8513031; doi:10.3390/molecules26195941)
Supplement: Supplementary file 1 [file molecules-26-05941-s001.zip › supplFigures-and-files-noArg-rev/supplFiles-1-to-8.pdf]

Supplementary File 1
Quality check for wtGALT at 310K

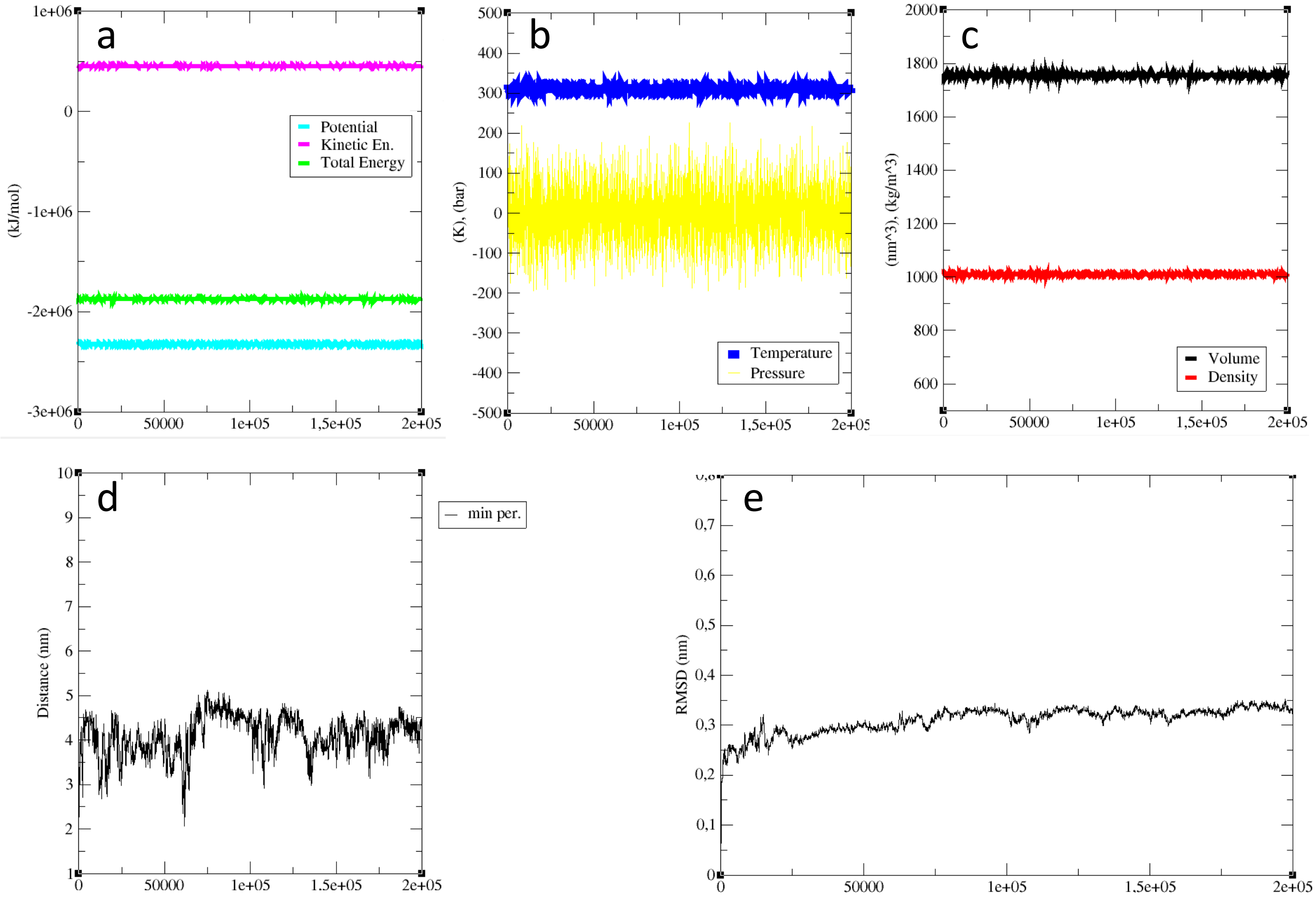

Legend: a: evolution of potential, kinetic and total energy; b: evolution of temperature and pressure; c: evolution of volume and density; d: variation of minimum distance between periodic images; e: root mean square deviation (RMSD) of atom distances..

Supplementary File 2
Quality check for p.Gln188Arg at 310K

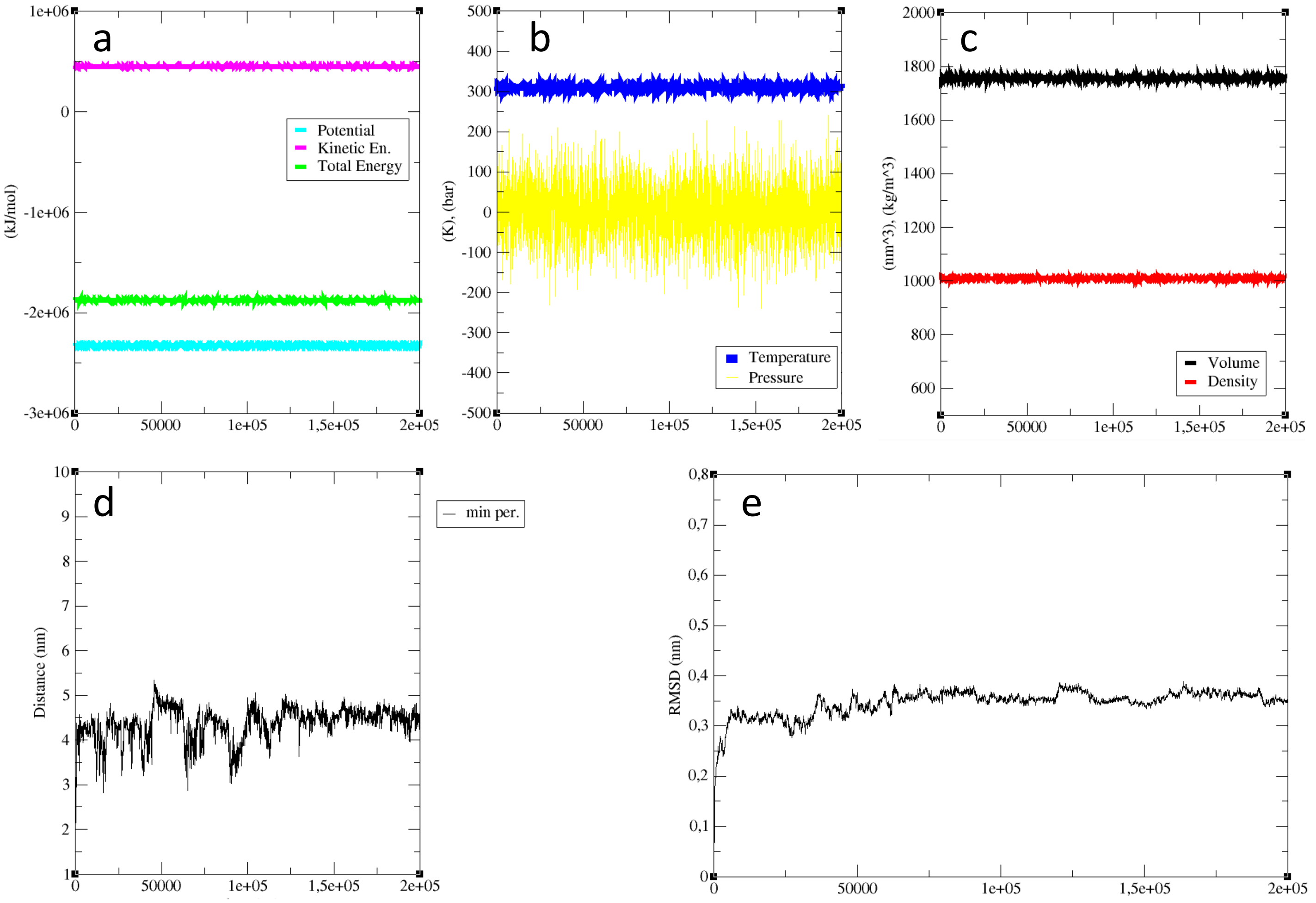

Legend: a: evolution of potential, kinetic and total energy; b: evolution of temperature and pressure; c: evolution of volume and density; d: variation of minimum distance between periodic images; e: root mean square deviation (RMSD) of atom distances.

Supplementary File 3
Quality check for wtGALT + ligands at 310K

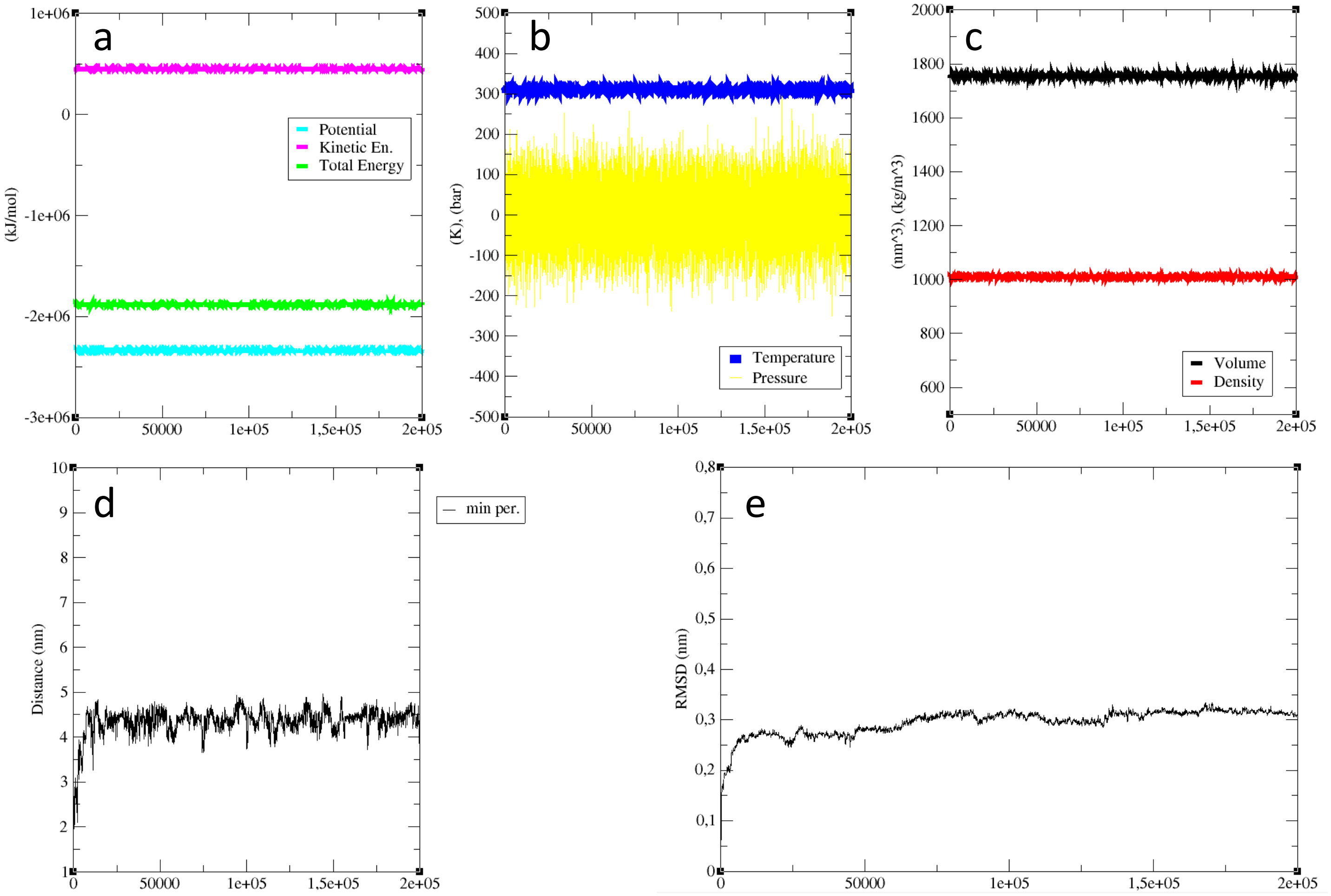

Legend: a: evolution of potential, kinetic and total energy; b: evolution of temperature and pressure; c: evolution of volume and density; d: variation of minimum distance between periodic images; e: root mean square deviation (RMSD) of atom distances.

Supplementary File 4
Quality check for p.Gln188Arg + ligands at 310K

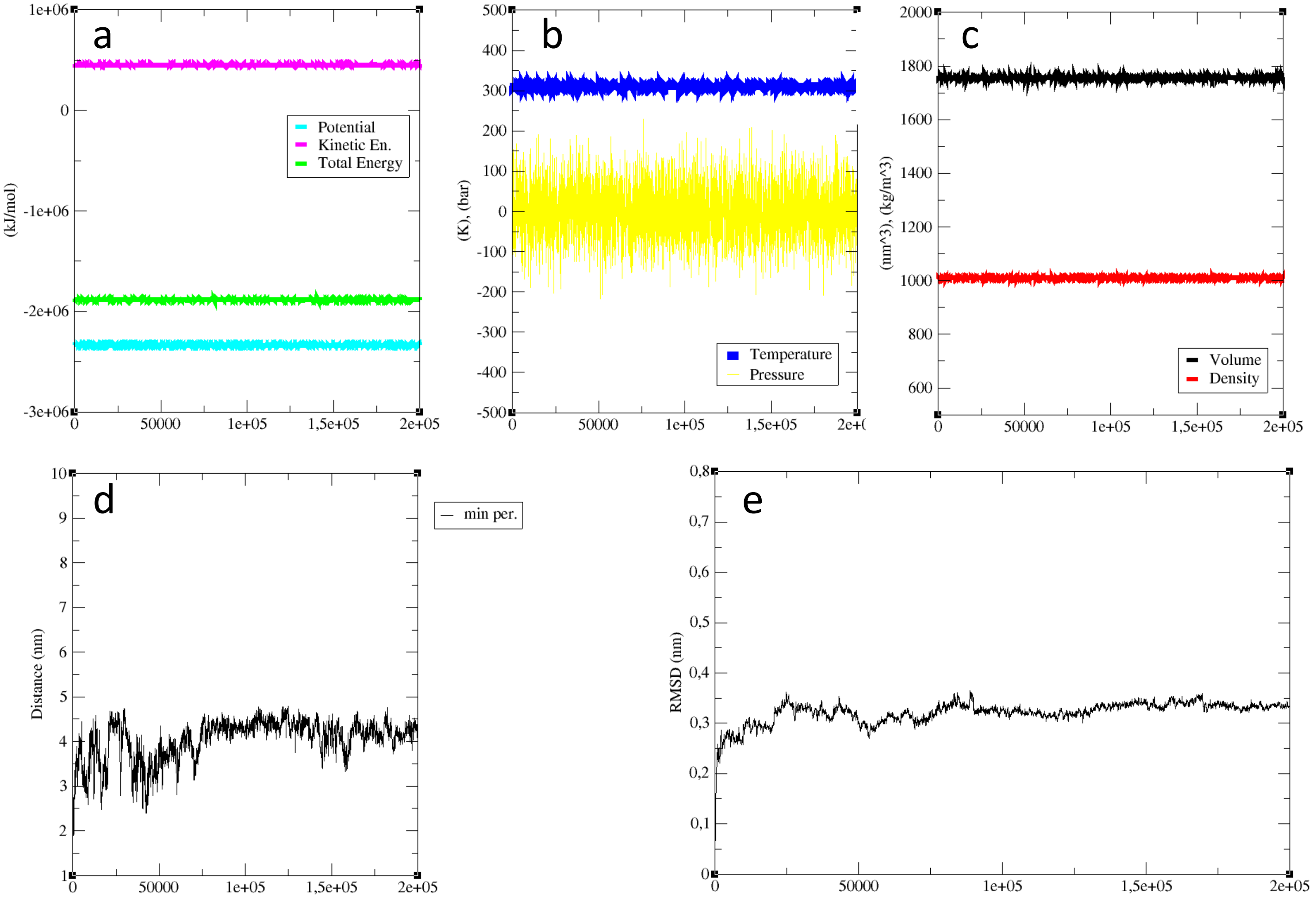

Legend: a: evolution of potential, kinetic and total energy; b: evolution of temperature and pressure; c: evolution of volume and density; d: variation of minimum distance between periodic images; e: root mean square deviation (RMSD) of atom distances.

Supplementary File 5
Quality check for wtGALT at 334K

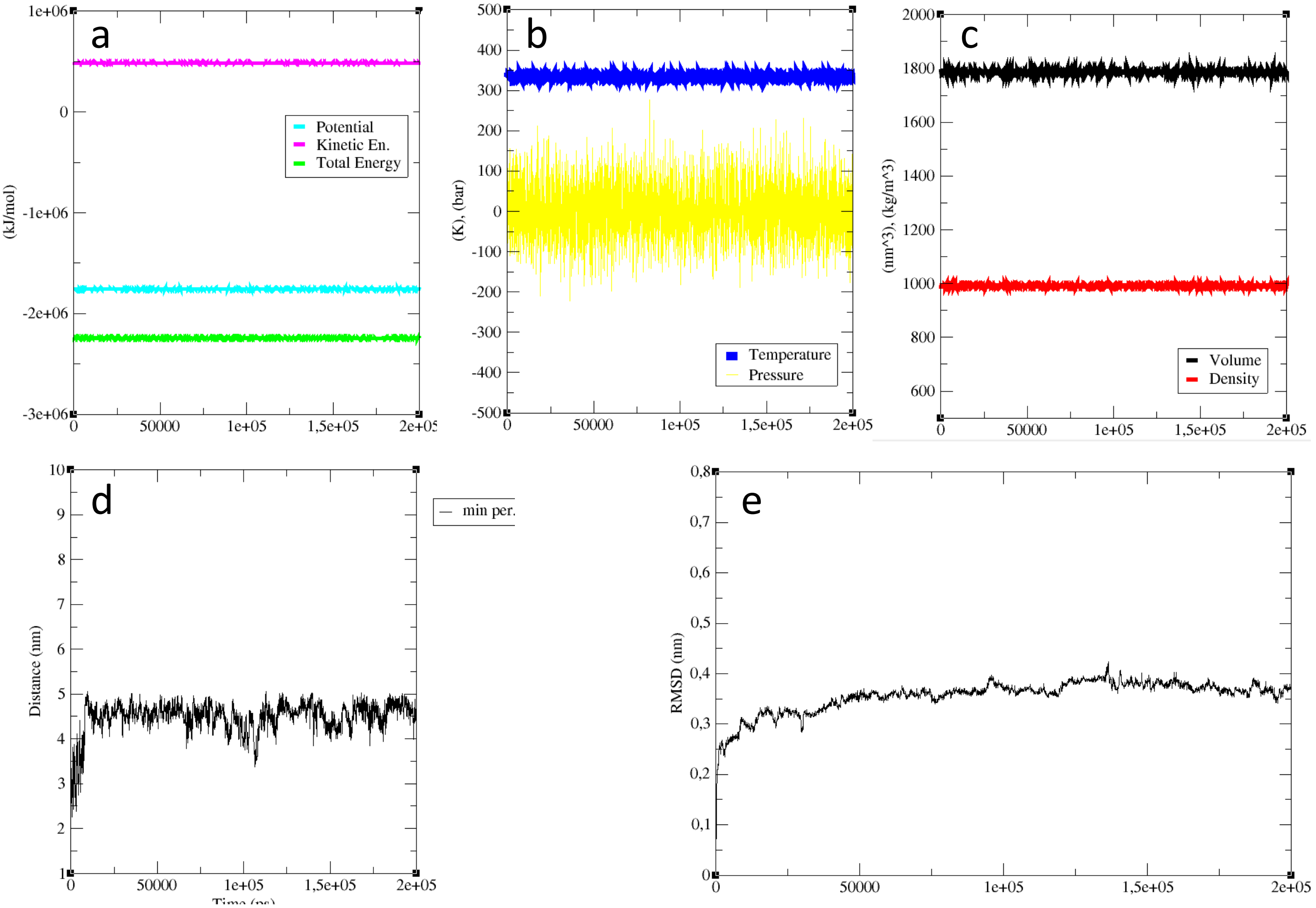

Legend: a: evolution of potential, kinetic and total energy; b: evolution of temperature and pressure; c: evolution of volume and density; d: variation of minimum distance between periodic images; e: root mean square deviation (RMSD) of atom distances.

Supplementary File 6
Quality check for p.Gln188Arg at 334K

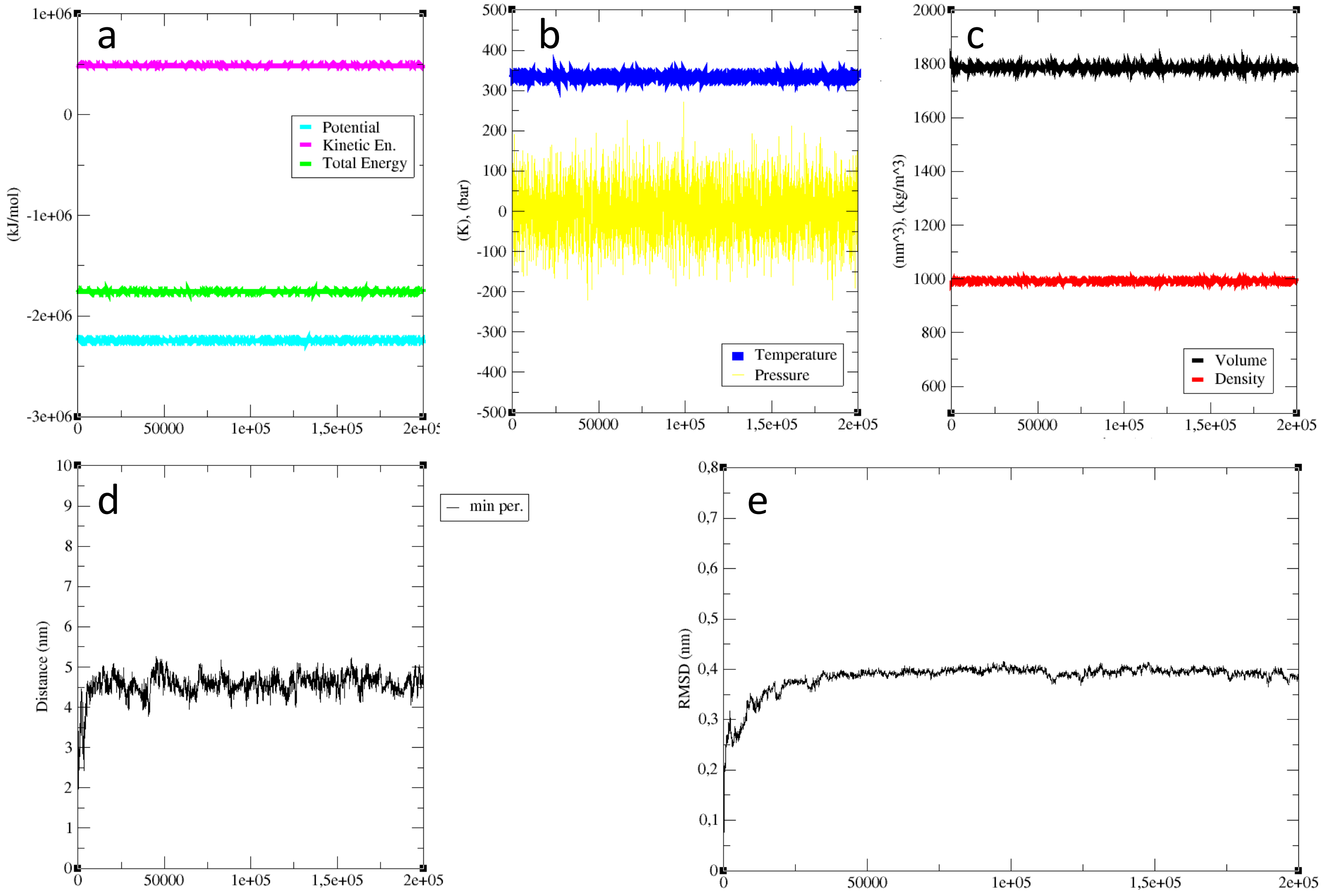

Legend: a: evolution of potential, kinetic and total energy; b: evolution of temperature and pressure; c: evolution of volume and density; d: variation of minimum distance between periodic images; e: root mean square deviation (RMSD) of atom distances.

Supplementary File 7
Quality check for wtGALT + ligands at 334K

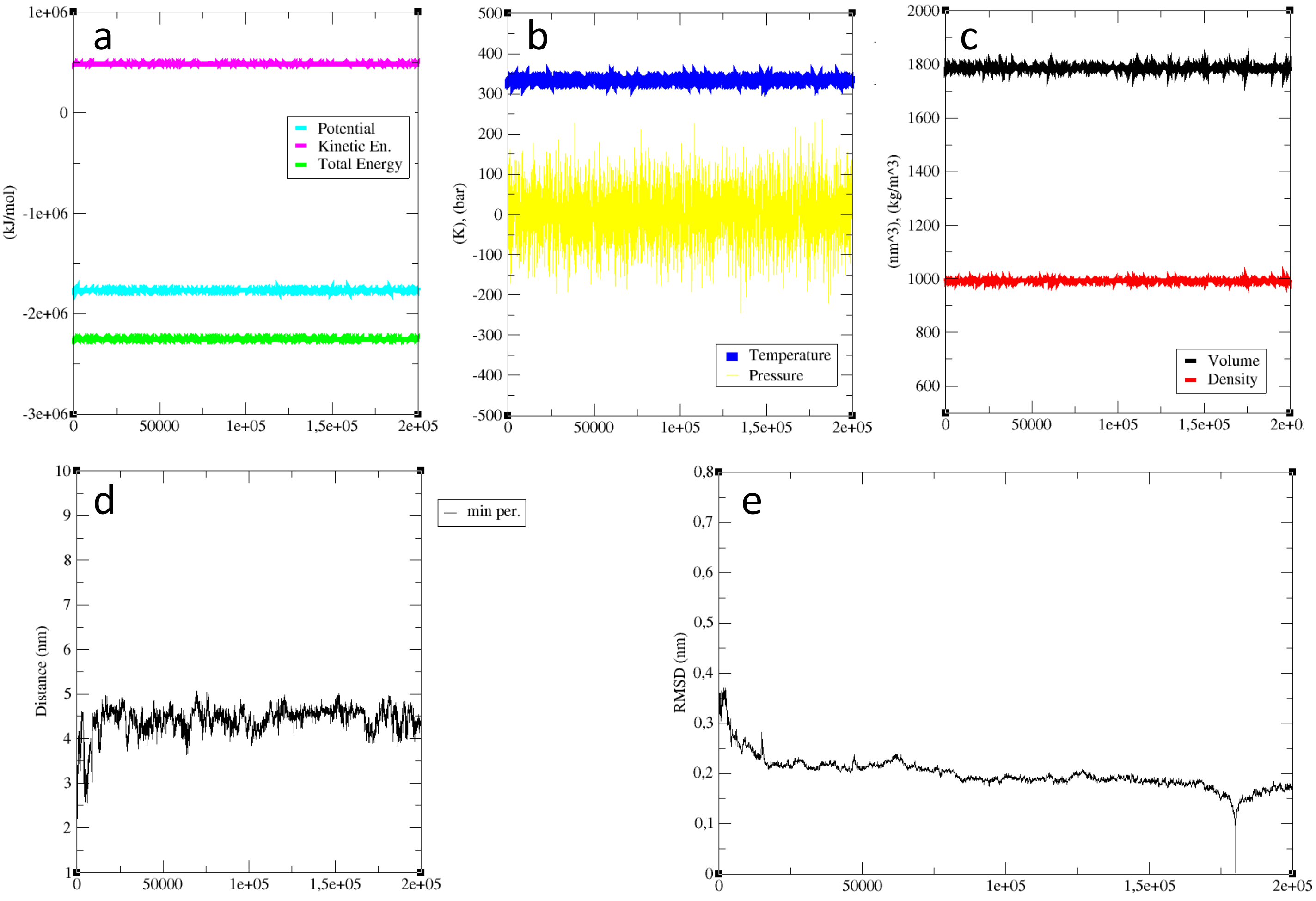

Legend: a: evolution of potential, kinetic and total energy; b: evolution of temperature and pressure; c: evolution of volume and density; d: variation of minimum distance between periodic images; e: root mean square deviation (RMSD) of atom distances.

Supplementary File 8
Quality check for p.Gln188Arg + ligands at 334K

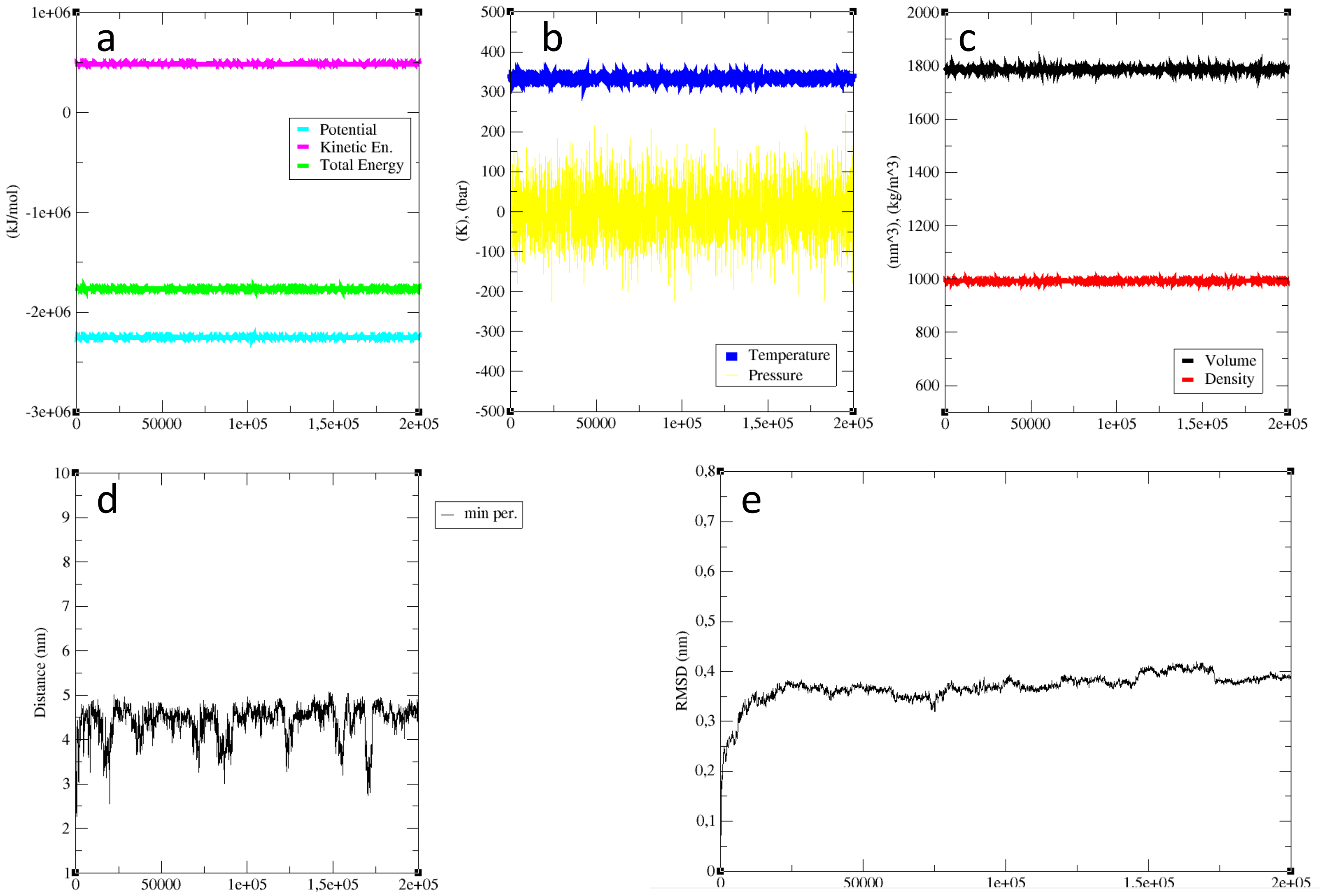

Legend: a: evolution of potential, kinetic and total energy; b: evolution of temperature and pressure; c: evolution of volume and density; d: variation of minimum distance between periodic images; e: root mean square deviation (RMSD) of atom distances.
