# Supplementary figures and images for: Analysis of the Structure-Function-Dynamics Relationships of GALT Enzyme and of Its Pathogenic Mutant p.Q188R: A Molecular Dynamics Simulation Study in Different Experimental Conditions
Source: Molecules. 2021 Sep 30;26(19):5941. doi: 10.3390/molecules26195941 (PMC8513031; doi:10.3390/molecules26195941)

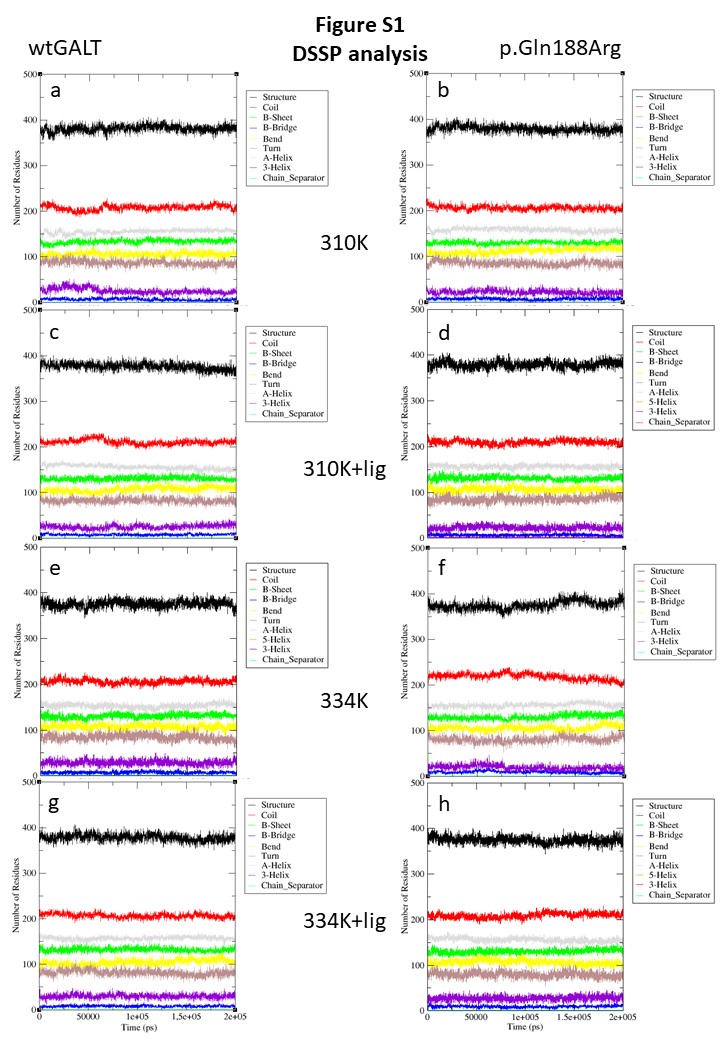

Supplement: Supplementary file 1 [file molecules-26-05941-s001.zip › supplFigures-and-files-noArg-rev/FigureS1.JPG]

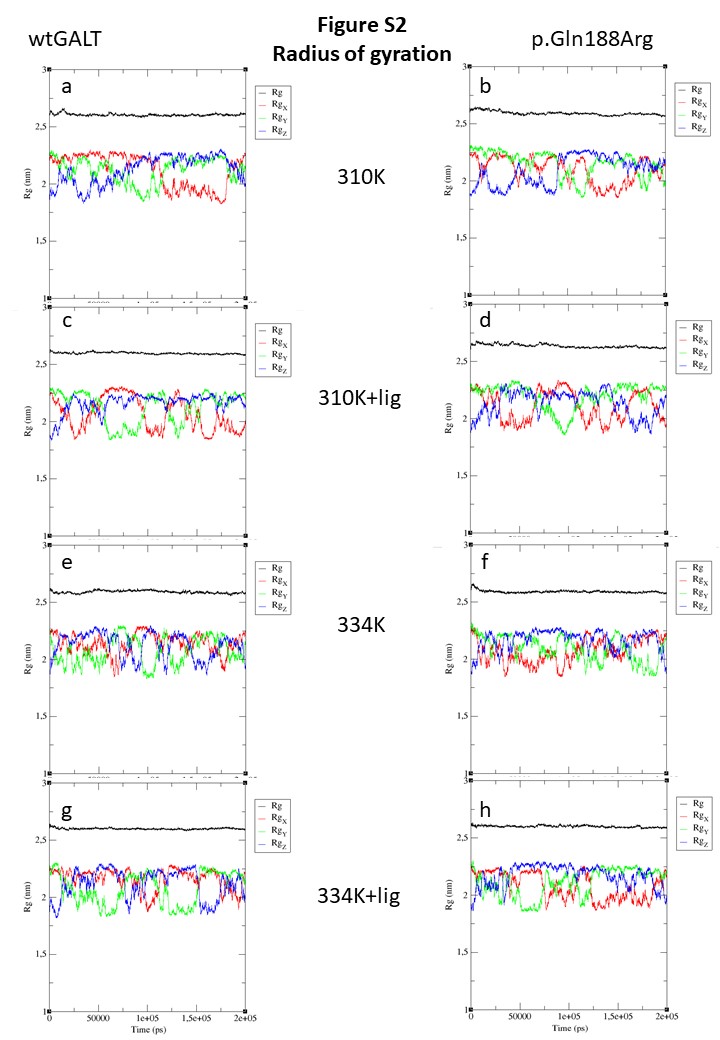

Supplement: Supplementary file 1 [file molecules-26-05941-s001.zip › supplFigures-and-files-noArg-rev/FigureS2.JPG]

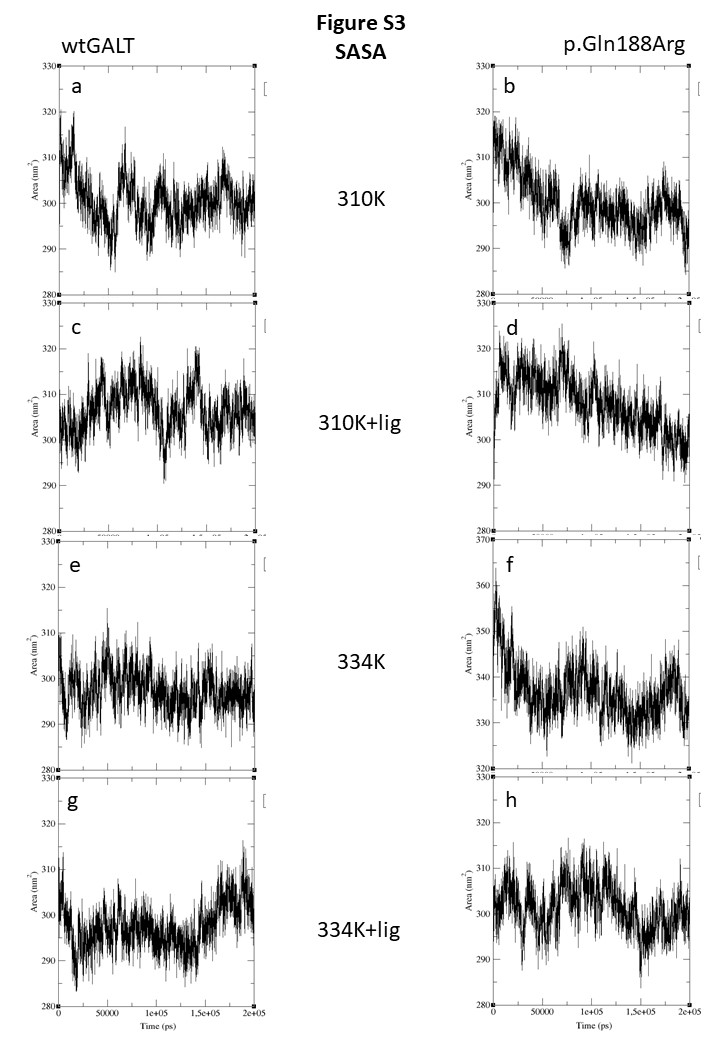

Supplement: Supplementary file 1 [file molecules-26-05941-s001.zip › supplFigures-and-files-noArg-rev/FigureS3.JPG]

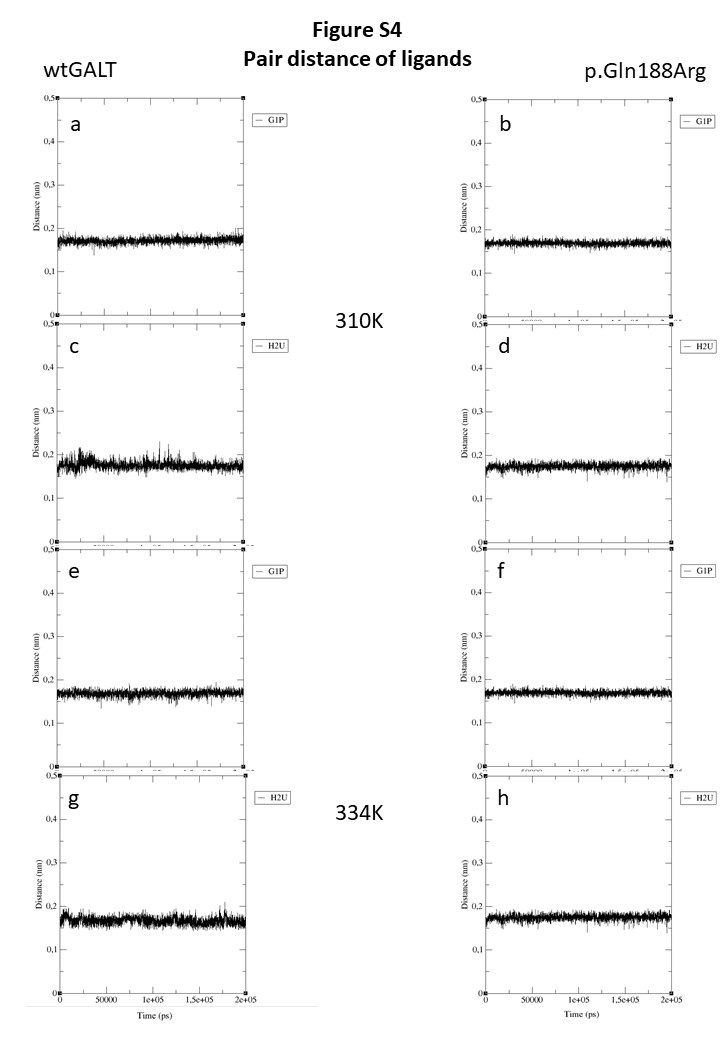

Supplement: Supplementary file 1 [file molecules-26-05941-s001.zip › supplFigures-and-files-noArg-rev/FigureS4.JPG]
